# INCREASE IN HSP25 EXTENDS LIFESPAN AND IMPROVES RESPONSE TO TAU TOXICITY THROUGH A CELL, NON-AUTONOMOUS MECHANISM

**DOI:** 10.1093/geroni/igz038.2678

**Published:** 2019-11-08

**Authors:** Karl Rodriguez

**Affiliations:** UT Health San Antonio, San Antonio, Texas, United States

## Abstract

The accrual of aggregation-prone cytotoxic proteins underlies neural pathologies seen in aging, Alzheimer’s disease and other dementias. Recent evidence indicates that heat shock protein 25kDa (HSP25) interacts with tau. To demonstrate a causal role for HSP25 in these pathologies, we overexpressed HSP25 protein in worms. This manipulation led to an increase in life span. Moreover, the longevity-effect was associated with increased expression of genes downstream of the SKN-1/Nrf2 stress-response transcription factor. HSP25 over-expression also reduces aggregate pathology and extends lifespan in a C. elegans neuronal-specific, aggregate-prone tau model . We propose that over-expression of HSP25 could provide protection from protein aggregation induced neurodegeneration. However, it is not yet clear whether this HSP25 effect could be efficaciously provided exogenously by other cell types. Thus, we will test whether increased peripheral HSP25 will reduce protein aggregation and stimulate a global Skn-1 stress-response pathway, reduce toxicity in neurons, and improve health outcomes.

